# Efficacy of cryodehydration technique in preserving the gross and histoarchitectural details of goat visceral and musculoskeletal specimens

**DOI:** 10.5455/javar.2023.j727

**Published:** 2023-12-31

**Authors:** Nasrin Sultana, Rafiqul Islam

**Affiliations:** Department of Anatomy and Histology, Bangladesh Agricultural University, Mymensingh, Bangladesh

**Keywords:** Cryodehydration, goat, cavitary organ, parenchymatous organs, musculoskeletal specimen

## Abstract

**Objective:**

This study sought to determine the effectiveness of the cryodehydration technique in preserving the morphologic and morphometric attributes of the anatomical specimens of goats.

**Materials and methods:**

Different anatomical parts of a goat, i.e., heart, lungs, spleen, liver, kidney, and musculoskeletal specimens, were collected and fixed in 15% formalin. Later on, the fixed specimens were cryodehydrated by fast freezing (burning process) and repeated freezing-thawing sessions, followed by wood glue coating. Finally, the macroscopic (i.e., weight, color, texture, odor, and durability) and microscopic characteristics (by routine hematoxylin and eosin staining) of the cryodehydrated specimens were studied.

**Results:**

The resultant specimens produced excellent color and texture and were lightweight (60%–80% weight loss), soft, dry, odorless, durable, and easy to handle. The histoarchitectural details of the heart and skeletal muscle were well preserved, while some distinctive alterations were observed in the parenchymatous organs, i.e., breach in cellular integrity, loss of cell cytoplasm, loss of cytoplasmic and nuclear clarity, increased sinusoidal space, dilatation of the renal tubules, and reduction in glomerular size. Nevertheless, the basic histoarchitecture of each specimen was yet to be distinctly identifiable.

**Conclusion:**

The current study findings suggest that the cryodehydration technique can preserve gross anatomical features as well as histoarchitectural details and can be an effective tool for facilitating veterinary education and research.

## Introduction

Veterinary anatomy teaching is crucial for macroscopic and microscopic knowledge about the different organs and tissues of animals. Therefore, the animal cadaver as well as its different anatomical segments (either fresh or preserved) are major parts of both the teaching and learning of veterinary anatomy [1]. These allow the students as well as the researchers to study each structure from three-dimensional aspects, as studied in living beings [2]. However, the decay of biological specimens is a major challenge faced in this process. Therefore, a large number of animals are sacrificed each year worldwide to facilitate veterinary education [3–3]. Moreover, researchers also utilize various traditional tissue preservation methods to ensure the long-term preservation of specimens. Formalin-based preservation is the most popular technique, while some other chemicals, i.e., ethanol, zinc chloride, glycerine, Keyserling solution, sodium chloride solution, etc., and methods, i.e., plastination and its modified techniques, cryopreservation, etc., are also being used [3–3,6–11]. Chemical-based tissue preservation methods have limitations for their use in teaching and learning purposes due to their inability to be handled bare-handed, let alone their negative impacts on human health [5]. Most of the chemical preservatives are toxic, induce allergic reactions and headaches in the body, damage the skin and mucous membranes, and even develop asthmatic conditions [12–12]. Researchers developed the plastination technique as an alternative to traditional chemical-based tissue preservation to combat these problems [11]. But the high cost of establishing a plastination laboratory and the requirement of patented chemicals have limited its use in veterinary education in lower or middle-income countries like Bangladesh [3–3]. In this context, the cryodehydration technique of biological specimen preservation can be an effective alternative.

A very low-temperature freezing technique has been widely used for centuries to preserve food and food ingredients [15]. Cryodehydration is the key step of cryopreservation, where the animal cadaver as well as the anatomical specimens are dehydrated through repeated freezing and thawing, following formalin fixation [16]. It is a kind of dry preservation technique that produces very lightweight final specimens, free from any irritating odor, is easy to store and transport, and can be handled bare-handed [2]. The formalin solution used initially for tissue fixation is also removed in the next steps of washing and repeated freezing-thawing sessions, reducing the risks of chemical exposure-related health issues [2,^16^]. Microrupture of the cell wall is an important phenomenon in the cryodehydration process as it facilitates fluid drainage to accomplish proper dehydration [17]. During cryodehydration, freezing produces extra and intra-cellular ice crystals that become liquified at the thawing step and drain out thereafter. Larger ice crystals are formed during the slow freezing technique, and hence, the preferable mode is rapid freezing because ice crystals that are small in size cause minimum tissue damage [16–16]. The fast-freezing method also helps to maintain the size and tissue morphology close to the fresh ones [16,^17^]. Previous studies have reported excellent outcomes in preserving the sliced canine head using the cryodehydration technique [16]. The cavitary and parenchymatous organs and muscular tissues can also be preserved in a near-natural state, according to study reports [2,^16^,^20^].

It is noteworthy that the primary goal of biological specimen preservation is to study both the microscopic characteristics and the gross morphology of the specimen. However, the earlier studies focused only on the efficacy of the cryodehydration technique in preserving the gross morphological aspects of the specimens [2,^16^]. However, no investigation has yet been conducted on the efficacy of this technique in preserving the histoarchitectural details of different types of organs or tissues. Despite being an effective low-cost dry preservation technique, this is a comparatively new concept in Bangladesh that needs to be studied and adopted. Considering the aforementioned facts and research gaps in this field, the current study‘s goals were to ascertain the effectiveness of cryodehydration techniques in preserving the morphologic and morphometric attributes of the visceral and musculoskeletal specimens of goats from both the macroscopic and microscopic aspects.

## Materials and Methods

### Ethical statements

The research was carried out entirely in the Department of Anatomy and Histology, FVS, Bangladesh Agricultural University (BAU), Bangladesh. Ethical clearance for animal experimentation was obtained from Animal welfare and Experimentation Ethics Committee (AWEEC), BAU, Bangladesh [AWEEC/BAU/2023(44)].

### Collection of specimens

The biological specimens (i.e., heart, lungs, liver, kidney, spleen, and musculoskeletal specimens) were obtained from a healthy goat. The initial weight (g) of each specimen was recorded just after collection.

### Fixation and freezing

The collected specimens were fixed in a 15% neutral buffered formalin solution for one week to fix and harden the tissues. Washing of the fixed specimens was done under running water for 45 min to get rid of the excess formalin and placed in a freezer at −20°C for one week.

### Cryodehydration process

#### Burning phase

After freezing, the first phase of cryodehydration called the “burning phase” started, where the specimens were laid on a tray without any lid and kept in the freezer (at −20°C) till they were completely frozen. Then, the frozen specimens were thawed at room temperature (25°C) for 2 h without any heat treatment. To expose both sides of the specimens to frozen temperatures, each specimen was turned after each freezing-thawing session. This process was repeated every 24 h routinely. After each thawing session, the water was removed from the tray, and the surface water was soaked with a tissue napkin. Thus, the freezing-thawing was repeated twenty times for smaller specimens (i.e., heart, kidney, and spleen) and thirty to forty times for the larger specimens (i.e., liver, lungs, and musculoskeletal specimens) to complete the burning phase. The burning phase was assumed to be completed when the specimens attained a light surface color, fibrous texture, and sponginess.

#### Dehydration phase

To dehydrate the specimens, defrosting was done at room temperature (15–30 min at 25°C) and then kept in the freezer immediately after defrosting. The procedure was repeated until the specimens were completely dried.

### Painting with wood glue and storage

The cryodehydrated specimens were then weighed (final weight) and painted with wood glue using a soft brush to give them a protective coating over the surface to prevent any moisture absorption. Once the glue was dried, the specimens were stored in airtight plastic containers with silica gel. The weight losses of the specimens were determined using the following equation:

$Loss of weight (\%)=\frac{\text{ Initial weight-Final weight }}{\text{ Initial weight }}\times 100$ .

### Microscopic investigation

To study the microscopic characteristics of the cryodehydrated specimens, approximately a 0.5-square-cm sample was taken from each specimen (both fresh and cryodehydrated) and processed for routine staining (Harris hematoxylin and eosin stain) following the standard protocol [21]. The histomorphological and histomorphometric characteristics (i.e., the thickness of the heart wall, the circumference of the bronchi and bronchioles, the surface area of lung alveoli, the circumference of the hepatic central veins (CVs) of the liver and renal tubules (RT), the glomerular surface area, and the cross-sectional area of the MF) were investigated using a photomicroscope (Model: B-293, Optika, Italy).

### Statistical analysis

Analysis of all the numerical data gathered from the present investigation was performed using SPSS (version 22). The Shapiro–Wilk test was performed to analyze the homogeneity of the dataset. The differences in mean values were analyzed by a paired sample t-test, where *p* < 0.05 and *p* < 0.01 were considered significant and highly significant, respectively. The datasets were presented as mean ± SEM.

**Figure 1.**
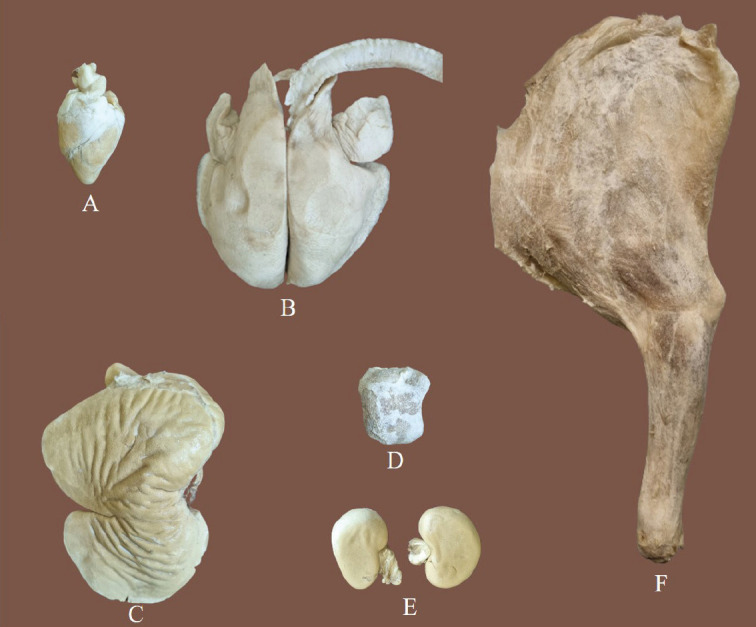
Gross appearances of the cryodehydrated specimens (A—heart, B—lungs, C—liver, D—spleen, E—kidney, F—musculoskeletal specimen (left forelimb).

## Results

### Macroscopic features of the cryodehydrated specimens

#### Morphologic features

The gross appearances of the cryodehydrated specimens are presented in Figure 1. The cavitary organ (i.e., heart) required a lesser (~20 cycles) freezing-thawing cycle compared to the parenchymatous organs (~30 cycles) and musculoskeletal specimens (~40 cycles). The cryodehydrated specimens produced excellent color and texture and retained their morphological aspects in near-natural states. They were soft, dry, odorless, lightweight, and durable (stored at room temperature for 3 months without any visible alteration). No shrinkage occurred in any of the organs except the liver, which had noticeable shrinkage on its parietal surface only.

#### Morphometric features

The morphometric attributes (initial weight, final weight, and percentage of weight loss) of the organs are shown in Table 1. The data show that the final weight of each organ subjected to cryodehydration was substantially reduced. The maximum percentage of weight loss was found in the case of the lungs, while the musculoskeletal specimen (left forelimb) had the minimum percentage of weight loss. This implies that the cryodehydrated specimens are very lightweight and easy to handle. Overall, the cryodehydration process resulted in 60%–80% weight loss in the final specimens.

### Microscopic features of the cryodehydrated specimens

#### Heart

**Figure 2.**
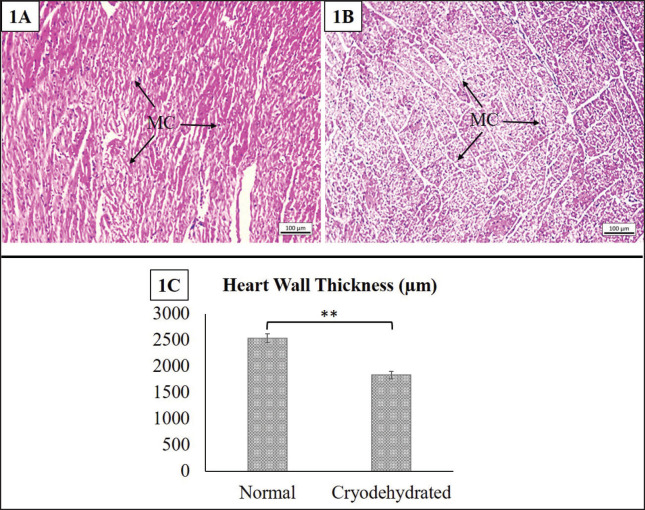
Histoarchitectures of the normal heart (1A) and cryodehydrated heart (1B) specimens. 1C presents the thickness of the heart wall of both the normal and cryodehydrated specimens. MC = myocardium. Magnification—100×, Scale bar—100 µm. Data are presented as mean ± SEM. The difference between normal and cryodehydrated specimens was compared using paired *t*-tests. **Difference between the mean values is highly significant (*p* < 0.01).

The histomorphological and histomorphometric features of the heart are presented in Figure 2. No distinct histoarchitectural alteration was noticed in the heart. All the muscle layers (i.e., epicardium, myocardium, and endocardium) were distinctly identifiable. However, the cytoplasmic clarity of the cardiac muscle was slightly lost. A noticeable alteration in the heart wall thickness was found in the histomorphometric investigation. The heart wall thickness was markedly reduced (*p* = 0.002) in comparison to the normal heart, indicating the shrinkage of the myofibers (MF) following cryodehydration.

**Table 1.** Initial weight (fresh specimens), final weight (cryodehydrated specimens), and weight loss (%) of heart, lungs, liver, kidney, spleen, and musculoskeletal specimens.

Organ weight	Initial weight (gm)	Final weight (gm)	Weight loss (%)
Heart	73	16	78.08
Lungs	167	35	79.04
Liver	384	83	78.39
Kidney	49	12	75.51
Spleen	23	6	73.91
Forelimb (left)	657	251	61.80

#### Lungs

**Figure 3.**
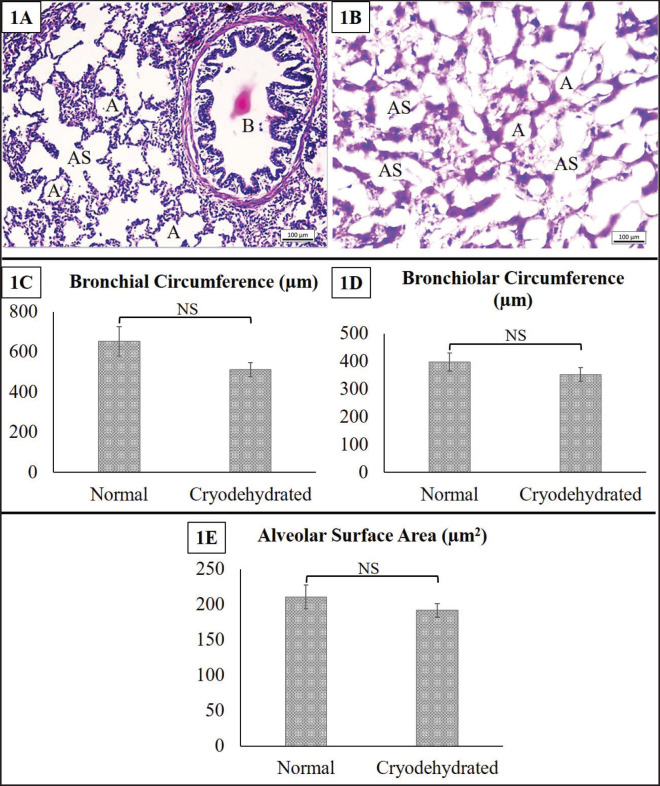
Histoarchitectures of the normal lungs (1A) and cryodehydrated lungs (1B) specimens. 1C, 1D, and 1E present the bronchial circumference, bronchiolar circumference, and alveolar surface area, respectively, of both the normal and cryodehydrated specimens. A = alveolus, AS = alveolar sac, and B = bronchiole. Magnification—100×, Scale bar—100 µm. Data are presented as mean ± SEM. The difference between normal and cryodehydrated specimens was compared using paired *t*-tests. NS = non-significant.

The histomorphological and histomorphometric characteristics of the lungs are shown in Figure 3. The histoarchitectural details of the lungs were quite altered following cryodehydration. However, the lung alveoli and alveolar sacs were yet distinctly identifiable. Even though no substantial change (*p* > 0.05) was observed in the histomorphometric investigation, all the histomorphometric attributes, i.e., bronchial circumference (*p* = 0.185), bronchiolar circumference (*p* = 0.157), and alveolar surface area (*p* = 0.453), were reduced in the cryodehydrated lungs.

#### Spleen

**Figure 4.**
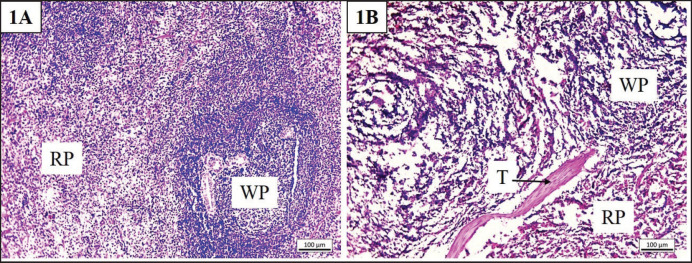
Histoarchitectures of the normal spleen (1A) and cryodehydrated spleen (1B) specimens. RP = red pulp, T = trabecula, and WP = white pulp. Magnification—100×, scale bar—100 µm.

The histomorphological features of the spleen are shown in Figure 4. The histoarchitectural details of the spleen were lost to some extent with the loss of cellular integrity. However, the zones of red pulp and white pulp were still distinguishable with distinct trabecular septa.

#### Liver

**Figure 5.**
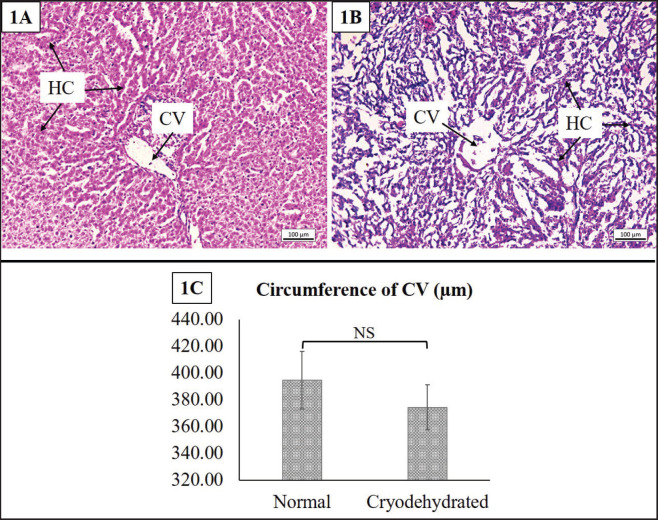
Histoarchitectures of the normal liver (1A) and cryodehydrated liver (1B) specimens. 1C presents the circumference of the CV of both the normal and cryodehydrated specimens. Magnification—100×, Scale bar—100 µm. Data are presented as mean ± SEM. The difference between normal and cryodehydrated specimens was compared using paired *t*-tests. NS = non-significant.

The histomorphological and histomorphometric attributes of the liver are presented in Figure 5. The loss of cytoplasmic detail in the hepatocytes was evident in the cryodehydrated liver. However, the hepatocytic nuclei were still intact. The hepatocytic cords were distinctly identifiable, and the sinusoids were found to be dilated. The histomorphometric investigation indicated no mentionable difference (*p* = 0.451) in the circumference of the CV, though it tended to decrease in the cryodehydrated liver compared to the normal liver.

#### Kidney

**Figure 6.**
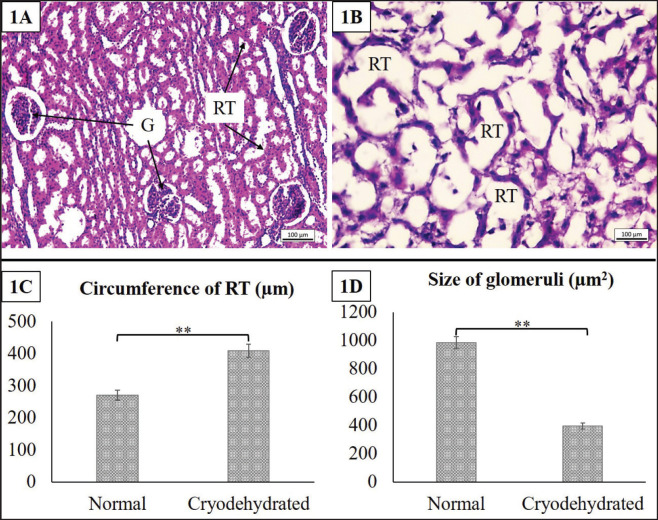
Histoarchitectures of the normal kidney (1A) and cryodehydrated kidney (1B) specimens. 1C and 1D represent the circumference of the RT and glomerular surface area of both the normal and cryodehydrated specimens. Magnification—100×, scale bar—100 µm. Data are presented as mean ± SEM. The difference between normal and cryodehydrated specimens was compared using paired *t*-tests. **Difference between the mean values is highly significant (*p* < 0.01).

The histomorphologic and histomorphometric characteristics of the kidney are given in Figure 6. In the histomorphologic investigation, some distinct alterations were noticed. The renal glomeruli were shrunk, even lost, in some areas. The RT were found intact but dilated. The cytoplasmic detail and nuclear clarity were also intact. However, these findings were further confirmed by histomorphometric investigation, which showed a significantly increased circumference of the RT (*p* = 0.001) while the size of the glomeruli markedly decreased (*p* = 0.0003).

#### Skeletal muscle

**Figure 7.**
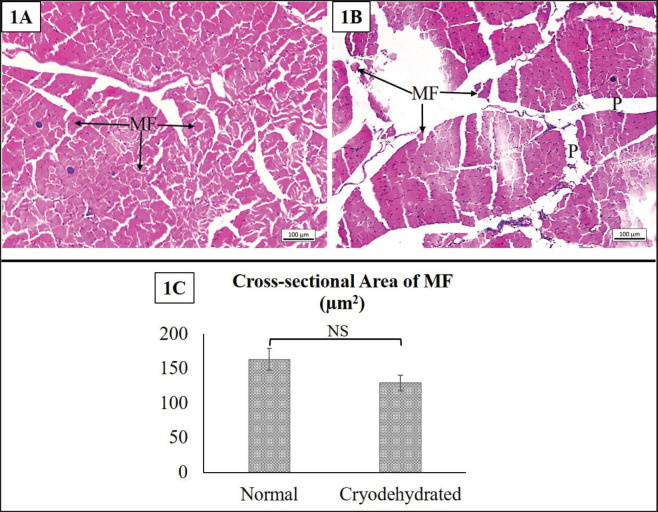
Histoarchitectures of the normal muscle (1A) and cryodehydrated muscle (1B) specimens. 1C presents the cross-sectional area of the MF of both the normal and cryodehydrated specimens. MF = myofibers and P = perimysium. Magnification—100×, scale bar—100 µm. Data are presented as mean ± SEM. The difference between normal and cryodehydrated specimens was compared using paired *t*-tests. NS = non-significant.

The histomorphologic and histomorphometric features of the skeletal muscle are presented in Figure 7. No distinct alteration in the histoarchitecture of the skeletal muscle was noticed, except for some localized loss of MF. The remaining MF were intact, with cytoplasmic and nuclear clarity. However, the space (where the perimysium is located) between the muscle bundles was increased. In the histomorphometric investigation, no mentionable difference (*p* = 0.257) in the cross-sectional area of the MF was found. However, the cross-sectional area of the MF tended to decrease, indicating the shrinkage of MF following cryodehydration.

## Discussion

A wide range of techniques have been used for centuries to preserve biological specimens for academic, research, and diagnostic purposes [4,^5^,^7^,^9^,^16^,^22^]. Researchers are still searching for an alternative tissue preservation technique that is simultaneously effective, health-safe, and cheaper. The cryodehydration technique is a modification of the methods first applied to preserve cavitary and parenchymatous organs [20]. This technique is now used to preserve anatomical segments, even whole-body sections, and cadavers [2,^16^].

### Macroscopic features of the cryodehydrated specimens

Varying degrees of weight loss in the cryodehydrated specimens were observed in comparison to their initial weight. Despite the weight loss and being lightweight, the cryodehydrated specimens produced excellent color and texture without any irritating or foul smell, preserving the specimens in near-natural states. These findings are in line with earlier reports where parenchymatous organs and body sections were subjected to cryodehydration to produce dry, light-weight specimens with no final odor [2,^16^]. One of the most prominent features of the cryodehydration technique is the loss of weight following dehydration. The water loss is necessary to prevent autolysis of the tissue components and microbial damage, ensuring the long-term preservation of cryodehydrated specimens [2,^5^,^9^,^16^]. The cavitary organ, like the heart, required less time to reach the final stage when compared with the parenchymatous organs, i.e., the lungs, liver, kidney, and spleen [2]. The hollow structure of the cavitary organs might be the reason behind requiring remarkably fewer freezing-thawing cycles to reach their final form. Noticeable variations were observed among the parenchymatous organs depending on their size, which supports the fact that the bigger the organ, the longer the period needed to obtain the final outcome [16]. Therefore, the sectioning or slicing of larger specimens into smaller ones produces even better final products within a shorter period of time [2,^16^]. According to an earlier report, sectioning or slicing might reduce the time duration required for cryodehydration by up to 25% [2]. Reduced sectioning or slicing of larger specimens also facilitates the study of the inner morphology of those organs [2,^16^,^19^]. The weight loss of different cryodehydrated specimens ranged between 60% and 80%, which is almost similar to the earlier reports [2,^16^]. It is noteworthy that almost similar results can also be obtained by using the plastination technique, despite requiring higher expenses to establish a plastination lab [4,^5^,^9^,^11^,^16^,^23^]. On the contrary, cryodehydration is a very simple and less expensive technique to prepare anatomical specimens for use in the classroom, exhibitions, and museums [2,^4^,^24^]. They are very durable and can be stored for up to 20 years, according to an earlier report [2]. Even though the current study results required the use of formaldehyde during the fixation step, its use was still remarkably reduced compared to traditional formalin preservation [24,^25^].

### Microscopic features of the cryodehydrated specimens

Preserving the structure is a critical criterion for using any kind of preservative or preservation technique [7,^17^,^23^]. There are very few research experiments that illustrate the efficacy of the cryodehydration technique in preserving different organs or anatomical structures from a macroscopic aspect. However, its efficacy in preserving the histoarchitectures of different organs is yet to be studied. Even though in the current study, the cryodehydration technique did not preserve the histoarchitectural details like the fresh ones, the minimal structural details necessary to identify an organ and to separate the basic structural components in tissue were preserved in this procedure. No distinct alteration in the heart or skeletal muscle was observed except for a massive decrease in the heart wall thickness and a non-significant decrease in the cross-sectional area of the MF, respectively. Cryodehydration results in 60%–70% water loss, which might be the underlying cause behind these alterations [2].

In the parenchymatous organs, the breach in cellular integrity, loss of cell cytoplasm as well as cytoplasmic and nuclear clarity, increased sinusoidal space, dilatation of the RT, and reduction in glomerular size were the major findings. Microrupture of tissue components is a common phenomenon in cryodehydration techniques [17]. Microrupture promotes the next stage of dehydration by causing gradual drainage of the intracellular and intercellular fluid and preventing significant shrinkage [16,^26^]. However, during the repeated freezing-thawing session, ice crystals are usually formed either within the cells or between the connective tissues and MF, resulting in cell wall rupture [16,^18^]. To combat this problem, fast freezing sessions are the best way to prepare cryodehydrated specimens, as they result in smaller ice crystal formation with minimal or no tissue damage [16,^26^]. During the dehydration phase, it also facilitates the fluid drainage required for tissue dehydration, avoiding mentionable tissue shrinkage [16,^17^,^19^]. If not, larger ice crystals form inside the cells or tissues that result in the breakdown of cellular structural integrity [15–15,26]. In addition, organs, tissues, capsules, and deep and superficial fascia all experience microruptures as a result of fluid dilatation. Therefore, the loss of cytoplasm as well as cellular integrity is justified. The increase in sinusoidal space and dilatation of RT might be due to the additional space created by the loss of extracellular fluid during the dehydration process [23]. However, the cellular damage can be minimized by a controlled thawing process where thawing is to be done at a temperature lower than room temperature to avoid tissue shock and prevent excessive osmotic gradient [17,^18^].

The weak points in the current study are that it only investigated the efficacy of cryodehydration techniques in preserving different organs and tissues of goats and did not use controlled thawing techniques during the dehydration phase. Therefore, interpretations cannot be made based on these findings in terms of preserving a full animal cadaver or organs that are more voluminous than goat. Another drawback of this technique is that it may take several weeks or months to prepare cryodehydrated specimens, especially in the case of larger specimens.

## Conclusion

The cryodehydrated specimens were very soft, dry, odorless, lightweight, durable, easy to handle, and comparatively safer than traditional chemical-based preservation methods. The major advantage of cryodehydrated specimens is their suitability to be used for teaching purposes, as they can be studied with bare hands. However, the use of cryodehydration techniques in preserving histoarchitectures is dependent on the type of specimen and purpose of the study. This technique can be used to identify and study the general histoarchitecture of an organ. Where a more detailed investigation of tissue components is necessary, a controlled thawing process is needed to be followed during repeated freezing-thawing sessions. Therefore, a further study is recommended to prepare cryodehydrated specimens following the controlled thawing method.

## List of abbreviations

AWEEC, Animal welfare and Experimentation Ethics Committee; BAU, Bangladesh Agricultural University; FVS, Faculty of Veterinary Science; gm, Gram.
